# Evaluation of knowledge and barriers of influenza vaccine uptake among university students in Saudi Arabia; a cross-sectional analysis

**DOI:** 10.7717/peerj.13959

**Published:** 2022-09-28

**Authors:** Tauqeer Hussain Mallhi, Nida Bokharee, Munnaza Bukhsh, Yusra Habib Khan, Abdulaziz Ibrahim Alzarea, Faiz Ullah Khan, Salah-Ud-Din Khan, Nasser Hadal Alotaibi, Abdullah Salah Alanazi, Muhammad Hammad Butt, Ahmed D. Alatawi, Muhammad Shahid Iqbal

**Affiliations:** 1 Department of Clinical Pharmacy, College of Pharmacy, Jouf University, Sakaka, Al-Jouf Province, Kingdom of Saudi Arabia; 2 Pharmacy Services Department, Midcity Hospital, Lahore, Punjab, Pakistan; 3 Department of Medicine, Foundation University and Medical College, Islamabad, Pakistan; 4 Health Sciences Research Unit, Jouf University, Sakaka, Al-Jouf Province, Kingdom of Saudi Arabia; 5 Department of Pharmacy Administration and Clinical Pharmacy, School of Pharmacy, Xi’an Jiaotong University, Xi’an, China; 6 Department of Biochemistry, College of Medicine, Imam Mohammad Ibn Saud Islamic University, Riyadh, Kingdom of Saudi Arabia; 7 Department of Medicinal Chemistry, Faculty of Pharmacy, Uppsala University, Uppsala, Sweden; 8 Department of Clinical Pharmacy, College of Pharmacy, Prince Sattam bin Abdulaziz University, Al-kharj, Kingdom of Saudi Arabia

**Keywords:** Influenza, Vaccine hesitancy, Flu, Vaccines, Students, Universities, Oubtreak

## Abstract

**Background:**

Influenza vaccine hesitancy is a significant threat to global maneuvers for reducing the burden of seasonal and pandemic influenza. This study estimated the vaccine uptake, barriers, and willingness for influenza vaccines among university students in Saudi Arabia.

**Methods:**

A cross-sectional survey was conducted among health science (HS) and non-health science (NHS) university students. A 31-item questionnaire was used to ascertain the vaccination rate, barriers, and willingness for the flu vaccine.

**Results:**

This study included 790 students (mean age: 21.40 ± 1.94 years), 246 (31.1%) from HS and 544 (68.9%) from NHS disciplines. About 70% did not take flu shots before the arrival of the winter. The mean knowledge score was 7.81 ± 1.96, where 20.4%, 67.6%, and 12% of respondents had good, moderate, and poor knowledge regarding flu vaccines. The relative importance index (RII) analysis showed a lack of recommendation from physicians (51.5%, RI ranked: 1) was a top-ranked barrier to vaccine uptake, followed by negative perceptions and accessibility issues. Only 36.6% of the participants were willing to get vaccinated every year, 70% were willing to receive a vaccine on their doctor’s recommendations, and 46% agreed to vaccinate if vaccines were freely available in the university. The knowledge, barriers, and willingness widely varied across students from two disciplines.

**Conclusions:**

Our analysis underscored low flu vaccine uptake among university students. In addition, the study participants’ knowledge was unsatisfactory, and they were less inclined to receive the flu vaccine in the future. Lack of recommendation from the physicians, negative perceptions towards the flu vaccine, and difficult accessibility were found as significant barriers to the vaccine uptake. A multidimensional approach at educational institutes to cover the knowledge gap and address the barriers curtailing the vaccination rate among students is recommended.

## Introduction

The influenza virus is responsible for a contagious and acute respiratory infection commonly known as seasonal flu or influenza, contributing substantially to the global disease burden (WHO, 2019a). Seasonal influenza can cause illness of mild to even fatal nature manifested by fever, dry cough, headache, muscular pain, malaise, and joint pain (Hayward et al., 2014). The World Health Organization (WHO) in 2017 reported 250,000 to 500,000 death toll across the globe due to influenza (GBD 2017 Influenza Collaborators, 2019). These estimates were revisited by Paget et al. (2019), in 2019, with an average of 389,000 deaths per annum. However, flu vaccine receipt reduces the risk of contracting the infection and the associated morbidity and mortality (Buchman et al., 2021). WHO recommends influenza vaccine uptake on an annual basis due to continuous genomic changes in the influenza virus (WHO, 2019a; Rodríguez-Blanco & Tuells, 2019; Kim, Webster & Webby, 2018).

Despite the recommendation, the rate of flu vaccine uptake, unfortunately, remains lower in various regions around the globe, *i.e.*, in UAE: 24.7%, Southern California: 43%, and Australia: 53.8%, particularly 9.1% in Saudi Arabia (Abu-Gharbieh et al., 2010; Rogers, Bahr & Benjamin, 2018; Salem et al., 2019; Oakley et al., 2021). Various investigations from Saudi Arabia reported vaccine uptake rates of 48% to 58% in medical students and 41% in health care workers (Milunic et al., 2010; Ali et al., 2007; Lee et al., 2012; Al-Tawfiq, 2012). Vaccine hesitancy is a major challenge in vaccine receipt and one of the top ten threats to global health (WHO, 2019b). WHO defines vaccine hesitancy as the refusal or delay in acceptance of vaccines regardless of the availability of vaccination services (MacDonald, 2015). Additionally, some well-documented barriers to vaccine uptake include unavailability, inaccessibility, lack of motivation and knowledge regarding the benefits of the flu shot. These aforementioned barriers keep the vaccine coverage below WHO recommendation, *i.e.*, 90% (Lee et al., 2012; Nichol, D’Heilly & Ehlinger, 2008; Bednarczyk et al., 2015; Merrill et al., 2010). Some additional barriers influencing the willingness include affordability, inaccessibility, inconvenience, false perception, lack of knowledge, and awareness (Choucair et al., 2021; Ryan et al., 2019; Alhawsawi et al., 2020; Silva, Bratberg & Lemay, 2021). However, due to reportedly rising outbreaks of different types of seasonal flu in Saudi Arabia, a proactive understanding of barriers and motivators of vaccine uptake by the university students is of paramount importance to curb the growing encumbrance of vaccine-preventable diseases (VPDs) (WHO, 2019b; Balkhy et al., 2004).

Previously conducted studies in Saudi Arabia included healthcare workers (HCWs), parents, and medical students’ population (Alkathlan et al., 2020; Alshammari et al., 2014). However, there has been a shift in decision-making trends from parents to students over the years. Additionally, students have an increased potential for contracting the infection due to close proximity, living in crowded dorms, continuous social interaction at the workplace and social events, and more potential to transmit while returning home. In general, there is a knowledge gap between health and non-health sciences students. Medical students have a better understanding of disease and are more likely to receive vaccines than non-medical students. Moreover, healthcare students are considered future healthcare professionals, and WHO also has priorities in the vaccine campaign on healthcare workers, along with elderly people and pregnant women (Hayward et al., 2014; Hamdan et al., 2021). However, non-health students are the larger subset of the student community.

On the contrary, there is a dearth of investigations ascertaining the perception of non-medical students towards influenza vaccination. We intended this study to gather data on the flu vaccine uptake rate and to ascertain and compare the barriers to the uptake of flu shot among the non-health sciences students and healthcare students of Jouf, Saudi Arabia. In addition, evaluating knowledge and perception regarding influenza vaccine among the two groups of students will aid the health authorities to design and implement vaccination campaigns in educational institutes. To the best of our knowledge, this study is novel in that aspect we included students from both health and non-health disciplines.

## Materials & Methods

### Ethical statement

Ethical approval was obtained from the Institutional Committee of Bioethics (Reference no.: 06-05-43). The participation in this study was on a volunteer basis and written informed consent was obtained from each participant. A brief description of the study and its purpose was also provided at the beginning of the study. The anonymity of the respondents was ensured before analysis.

### Study site and design

**Figure 1:**
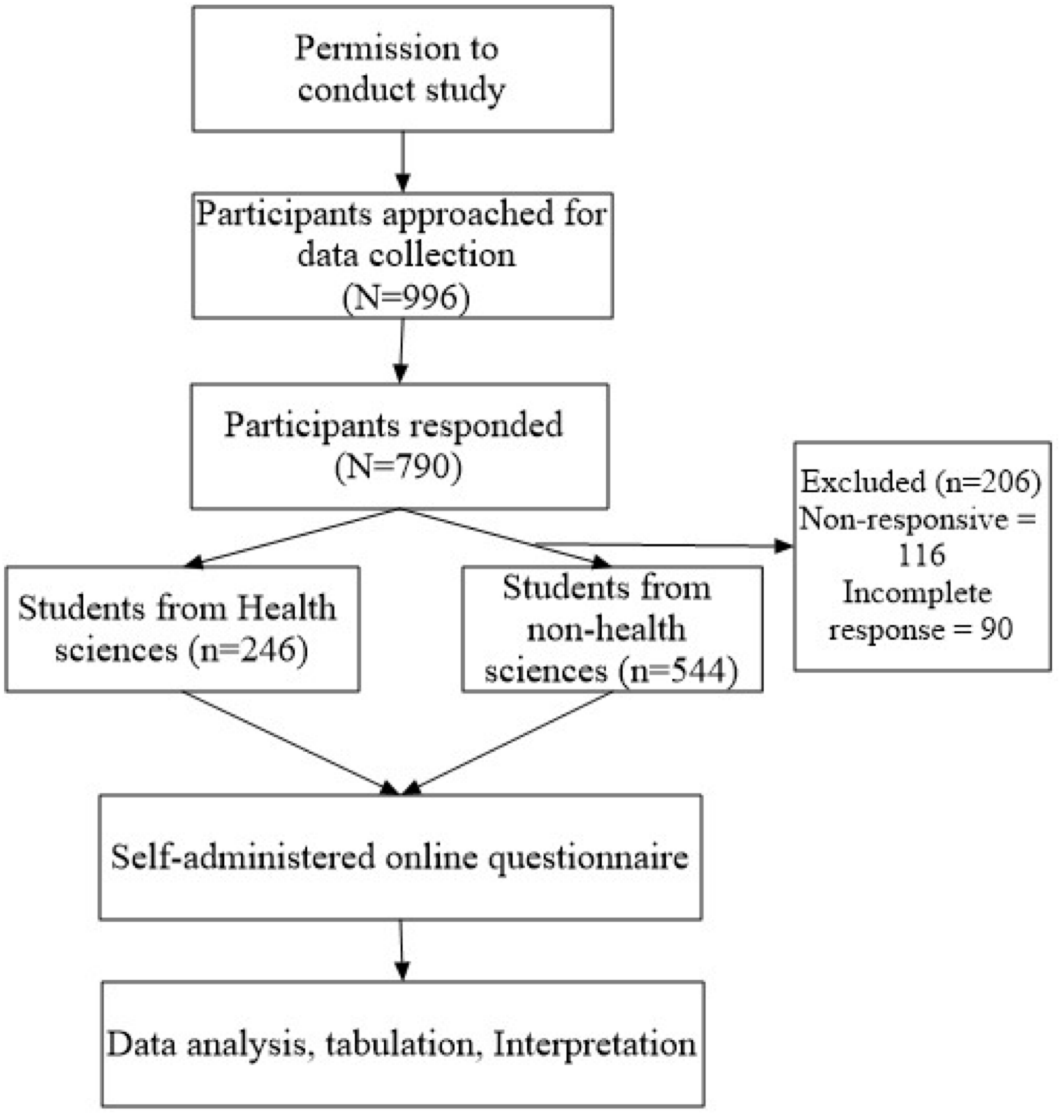
Study flow diagram.

This web-based cross-sectional study was conducted among students from Jouf University, Saudi Arabia. Inclusion criteria for recruitment into the present study were: (1) All the students enrolled in the university, regardless of their discipline and year of study, (2) willing to participate, (3) age 18 years and above, (4) either male or female, and (5) were able to write and read Arabic or English. All the respondents not fulfilling the inclusion criteria were excluded from the study. The methodological flow chart of the current study is described in Fig. 1.

### Study duration

Data was collected over five months period, starting from February to June 2021.

### Sample size and sampling technique

The minimum sample size was estimated using the Cochran formula for sub-groups in a population:

\begin{eqnarray*}n= \frac{2{Z}^{2}pq}{{d}^{2}} . \end{eqnarray*}

Where, *n* = desired sample size, *Z* = level of confidence according to standard normal deviation (1.96 corresponding to 95% confidence interval), *p* = proportion in the target population having a particular characteristic, *q* = proportion in the target population not having particular characteristics, *i.e.*, 1-p, and d = degree of accuracy required (0.05). The minimum population size was adjusted for non-response and drop-out of 25% with the power of 0.80, resulting in a required sample size of 424.

### Study instrument

A 31-items questionnaire was developed after reviewing previous literature (Rogers, Bahr & Benjamin, 2018; Benjamin & Bahr, 2016). The panel of experts reviewed the study instrument for the content and face validity, and their recommendations were considered in the finalized questionnaire. The validity of the questionnaire was determined by piloting a sample of 30 students to ascertain if the questions were valid and understandable. This data from the pilot study was not included in the final results. The reliability of the questionnaire was assessed by a Cronbach alpha value (0.77), which indicated the adequacy of the survey tool. The study instrument comprised of four sections:

1. Section I had questions related to demographics, including age, gender, marital status, the field of education, and questions related to flu vaccination status.
2. Section II consisted of thirteen true and false statements related to the knowledge of students about the flu vaccine. These items were 1 for “correct” answers and 0 for “wrong” and “don’t know” responses. The maximum cumulative knowledge score was 13. The knowledge score was further categorised into three levels, *i.e.*, poor knowledge (score ≤ 5), moderate knowledge (score 6 to 9), and good knowledge (score ≥ 10) (Salman et al., 2020; Srichan et al., 2020). Section III consisted of questions identifying the barriers influencing the receipt of the flu vaccine. Eleven barriers in this study were broadly classified under five categories. These five categories of barriers included affordability (1 item), accessibility (2 items), negative perception (5), lack of recommendation (1 item), and religious and cultural restrictions (2 items). All the responses were collected on a 5-point Likert scale (“Agree”, “Strongly agree”, “Neutral”, “Disagree”, to “Strongly disagree”). Responses were collapsed for further analysis by re-coding the values for strongly agree and agree as ’1”; the values for disagree, strongly disagree, and neutral as ’0”. The maximum barrier score was 11. Percentage agreement was calculated for the participants who agreed to the statements. Statements portraying the perceptions and barriers were ranked to determine the significant barriers associated with vaccine uptake (Bali et al., 2013; Eppes et al., 2013). Moreover, negative perceptions were specifically assessed in relation to vaccine uptake.
3. Section IV consisted of statements regarding the willingness of the students to take the flu shots. Willingness was determined using a set of seven questions with a response of “Yes”, “No” and “Don’t know”. These responses were re-coded and dichotomized, *i.e.*, 1 for agreeing to the statement and 0 for “disagree” and “don’t know” responses. The maximum willingness score was 7. The level of willingness was stratified into three levels: poor level (≤ 2 score), moderate level (3–5 score), and good level (≥ 6 score) of willingness to receive flu vaccine (Srichan et al., 2020; Eppes et al., 2013).

### Data collection

Convenient sampling was used and all the students were invited via electronic mail. Students’ e-mail addresses were obtained from the university database. Researchers approached the students via e-mails, with a link to the questionnaire enclosed with it. They explained the purpose of the survey, keeping the anonymity and confidentiality of the respondents. Incomplete responses were excluded from further analysis. The data collected online was scrutinised and converted to an excel sheet. Only completed responses were considered and subjected to further analysis. Survey responses were kept anonymous and were only accessible to the researchers.

### Operational definitions

Vaccinated participants were referred to those “who took the flu shot before the arrival of the winter season” and non-vaccinated were “those who did not receive it before winter”. Participants’ knowledge was stratified into three levels: Good, moderate, and low knowledge referred to knowledge score ≥10, 6-9, and ≤ 5, respectively. Barriers mean factors hindering vaccine uptake. Perception refers to participants’ beliefs regarding the vaccine uptake, whereas negative perception refers to the negative thinking of participants about the vaccine. The perception score is referred to as “the mean cumulative perception score”. Percentage agreement is “the % of participants who agreed to the statement or item of the study toll”. The participant was considered concerned if he/she agrees with the negative perception: extremely concerned, quite concerned, and little concerned. Vaccine acceptors referred to the “participants who agreed to take the vaccine every year”. Vaccine hesitant referred to the “participants who disagreed to take the flu vaccine every year”. Vaccine ambivalent referred to the “participants who were not sure to receive the flu vaccine every year”.

### Data analysis

Data analysis was done using SPSS version 21. Shapiro–Wilk and Kolmogorov–Smirnoff tests were used to evaluate the distribution of the data obtained from the survey. Continuous data were expressed as mean (±SD), while categorical data were summarised as frequency (%). Categorical data were compared using the Chi-square test or the Fisher exact test, where appropriate. The relative importance index (RII) was used to determine the pertinence of the statements, identifying and ranking the barriers to vaccine uptake. The value of RII ranges from 0 to 1; the value closest to 1 corresponds to the highest rank and main barrier. RII equation:

\begin{eqnarray*}RII= \frac{\sum W}{(A\ast N)} . \end{eqnarray*}

Where, N is the total number of participants, A is the highest weight, and W is the weight given to each statement by the participant (1: strongly disagreed to 5: strongly agreed). Furthermore, percentage agreement was calculated to evaluate the % of people who agree with the statement. To evaluate the % agreement to the negative perception statements, the 5-point Likert scale was collapsed to two points *i.e.*, “agree” and “disagree”. Mean knowledge and perception score were compared between the two groups using an independent *t*-test. Association between demographic variables, knowledge, perception, and willingness with vaccine uptake were evaluated using Pearson correlation, simple linear regression, or chi-square, where appropriate. Multivariate analysis using a logistic regression model was run to determine the association between the independent demographic variables with the flu vaccine uptake. The variables having a statistically significant value of 0.05 were included in the bivariate analysis. These predictive variables that showed significant association (*p*-value <0.05) on bivariate analysis were further included in a multivariable logistic regression model. Variables included in the model were age, gender, marital status, the field of education, knowledge, perception, and willingness. A 95% confidence interval (CI) was calculated to reveal the odds ratio (OR). *P*-value of <0.005 was considered statistically significant.

## Results

### Characteristics of study participants

Of the total 790 participants, 246 (31.1%) belonged to health sciences, and 544 (68.9%) were from non-health science disciplines. The mean age of participants was 21.40 ± 1.94 years, and most (83.9%) were single. There was an almost equal distribution of males (401, 50.8%) and females (389, 49.2%) gender. Table 1 summarises the demographic characteristics of the study participants.

**Table 1:** Demographic characteristics of the study participants.

**Variables**	**Total participants** **(*N* = 790)**	**Health sciences** **(*n* = 246)**	**Non-health sciences (*n* = 544)**	*p*-value
**Age** (years ± SD)	21.40 ± 1.94	21.87 ± 2.08	21.19 ± 1.84	**<0.001**
**Age categories**				<**0.001**
18–21 years	395 (50%)	99 (40.2%)	296 (54.4%)	
22–25 years	373 (47.2%)	136 (55.3%)	237 (43.6%)	
≥ 25 years	22 (2.8%)	11 (4.5%)	11 (2%)	
**Gender:***n* (%)				0.095
Male	401 (50.8%)	114 (46.3%)	287 (52.8%)	
Female	389 (49.2%)	132 (53.7%)	257 (47.2%)	
**Marital status:***n* (%)				**0.001**
Married	127 (16.1%)	56 (22.8%)	71 (13.1%)	
Single	663 (83.9%)	190 (77.2%)	473 (86.9%)	
**Years in university;***n* (%)				<**0.001**
1st year	104 (13.2%)	16 (6.5%)	88 (16.2%)	
2nd year	125 (15.8%)	34 (13.8%)	91 (16.7%)	
3rd year	235 (29.7%)	58 (23.6%)	177 (32.5%)	
4th year	227 (28.7%)	78 (31.7%)	149 (27.4%)	
5th year	74 (9.4%)	41 (16.7%)	33 (6.1%)	
More than 5 years	25 (3.2%)	19 (7.7%)	6 (1.1%)	
**Received flu vaccine before joining the university;***n* (%)				**<0.001**
Yes	338 (42.8%)	145 (58.9%)	193 (35.5%)	
No	452 (57.2%)	101 (41.1%)	351 (64.5%)	
**Received flu vaccine before winter season;***n* (%)				**0.001**
Yes	241 (30.5%)	95 (38.6%)	146 (26.8%)	
No	549 (69.5%)	151 (61.4%)	398 (73.2%)	
**Received flu vaccine during anytime in childhood;***n* (%)				0.446
Yes	533 (67.5%)	170 (69.1%)	363 (66.7%)	
No	66 (8.4%)	16 (6.5%)	50 (9.2%)	
Maybe	191 (24.2%)	60 (24.4%)	131 (24.1%)	

**Notes.**

SD Standard deviation

*p*-values were estimated using *t*-tests and *χ*^2^ test with a significance level of 0.05; these statistically significant values are in bold fonts in this table.

### Vaccination rate among study participants

The majority of the participants (*n* = 549, 69.5%) did not receive a flu shot before the winter season. There was a significant difference among the health sciences (HS) and non-health sciences (NHS) group w.r.t receipt of flu jab (flu shot before joining university: (HS:58.9% vs. NHS:35.5%; *p* < 0.001); and before arrival of the winter: (HS:38.6% vs. NHS:26.8%; *p* = 0.001)).

### Knowledge regarding influenza vaccination

One-third (67.6%) of study participants had moderate knowledge about the influenza vaccination with a mean knowledge score of 7.81 ± 1.96, differing significantly among the HS and NHS group (*p* < 0.001). Surprisingly, only 20.4% of students indicated good knowledge, with a majority being from the HS group (*n* = 88, 54.7%) (Table 2). Furthermore, high knowledge score was associated with gender (*p*-value < 0.001), field of education (*p*-value < 0.001), and year of education (*p*-value < 0.001). The knowledge score significantly differed across vaccinated and non-vaccinated participants (*p*-value 0.015; *F* = 3.152). Interestingly, females scored higher than males (8.48 ± 1.81 vs. 7.17 ± 1.88; *p* < 0.001). Item-wise analysis showed that most of the participants *correctly*responded that vaccines should be given to all persons aged six months and greater (*n* = 605, 76.6%), the vaccine reduces the severity and duration of flu (*n* = 576, 72.9%), and improves the immunity (*n* = 575, 72.8%).

**Table 2:** Knowledge among study participants regarding influenza vaccination.

**Variable**	**Total Correct response**	**Health sciences**		**Non-health sciences**		***p*-value**
		**Correct response**	**Incorrect response**	**Correct response**	**Incorrect response**	
	*n* (%)	*n* (%)	*n* (%)	*n* (%)	*n* (%)	
All persons aged 6 months and above should get influenza vaccination annually	605 (76.6%)	174 (70.7%)	72 (29.3%)	431 (79.2%)	113 (20.8%)	**0.009**
Influenza vaccination causes mild flu like symptoms	540 (68.4%)	201 (81.7%)	45 (18.3%)	339 (62.3%)	205 (37.7%)	**<0.001**
Being vaccinated reduces the severity and duration of flu	576 (72.9%)	216 (87.8%)	30 (12.2%)	360 (66.2%)	184 (33.8%)	**<0.001**
Being vaccinated, improves immunity	575 (72.8%)	193 (78.5%)	53 (21.5%)	382 (70.2%)	162 (29.8%)	0.016
Infants and immuno-compromised population cannot get influenza vaccination	430 (54.4%)	147 (59.8%)	99 (40.2%)	283 (48%)	261 (52%)	**0.043**
The complications of influenza can be severe leading to absence from schools and work place, effecting quality of work	431 (54.6%)	135 (54.9%)	111 (45.1%)	296 (54.4%)	248 (45.6%)	0.903
Severe influenza can lead to hospitalization and even death	439 (55.6%)	155 (63%)	91 (37%)	284 (52.2%)	260 (47.8%)	**0.005**
Influenza vaccine provides coverage for all types of strains that cause flu	357 (45.2%)	74 (30.1%)	172 (69.9%)	283 (52%)	261 (48%)	**<0.001**
Influenza vaccine reduces the severity and duration of flu for all types of strains	437 (55.3%)	162 (65.9%)	84 (34.1%)	275 (50.6%)	269 (49.4%)	**<0.001**
Influenza vaccine is not effective if I already got flu	393 (49.7%)	106 (43.1%)	140 (56.9%)	287 (52.8%)	257 (47.2%)	0.012
There are two types of influenza vaccine; intramuscular shot, intra nasal spray	459 (58.1%)	175 (71.1%)	71 (28.9%)	284 (52.2%)	260 (47.8%)	**<0.001**
The intramuscular influenza “shot” vaccine contains inactivated (killed) virus	483 (61.1%)	190 (77.2%)	56 (22.8%)	293 (53.9%)	251 (46.1%)	**<0.001**
The intranasal influenza “spray” vaccine (FluMist) contains live attenuated virus	443 (56.1%)	190 (77.2%)	56 (22.8%)	253 (46.5%)	291 (53.5%)	**<0.001**
**Knowledge score**						
Mean ± SD^*^	7.81 ± 1.96	8.75 ± 1.85		7.39 ± 1.86		**<0.001**
**Knowledge categories**						
Good knowledge score	161 (20.4%)	88 (54.7%)		73 (45.3%)		**<0.001**
Moderate knowledge score	534 (67.6%)	144 (27%)		390 (73%)		
Poor knowledge score	95 (12%)	14 (14.7%)		81 (85.3%)		

**Notes.**

Knowledge score out of 13.

* SD, Standard deviation

*p*-values were estimated using *χ*^2^ and *t*-tests with a significance level of 0.05; these statistically significant values and recorded in bold fonts in this table.

### Barriers to receiving flu vaccine

Our analysis compared 11 potential barriers to flu vaccine uptake (Table 3). Of these, “lack of recommendation by the doctor” was the highest-ranked (RI: 0.775), reported by 281 (51.5%) participants. The self-limiting nature of flu was ranked second (RI: 0.762), where 49.4% of participants responded that “flu is seasonal and will recover on its own”. Approximately half of the participants (50.3%) agreed with the statement “I believe that flu vaccine causes flu and fever” and ranked third (RI: 0.760). However, the lowest-ranked barrier was “vaccines are expensive,” and only 11.5% of participants agreed with it. Moreover, negative perceptions among participants were specifically assessed for their relationship with the vaccine uptake. The mean negative perception score was higher in those who did not receive the vaccine (5.17 ± 2.11; 68.5%).

**Table 3:** Potential barriers associated with the uptake of influenza vaccination.

**Potential barrier**	**% Agreement** ^a^			**RII** ^b^	**Ranked** ^c^
	**Total (*N* = 790)**	**Health sciences (*n* = 549)**	**Non-health sciences (*n* = 240)**		
**Affordability**					
Vaccines are expensive	121 (15.3%)	54 (44.6%)	67 (55.4%)	0.528	11
**Accessibility**					
I do not have time to get a flu vaccination	262 (33.2%)	81 (30.9%)	181 (69.1%)	0.639	7
I do not know where to receive a flu vaccination	220 (27.8%)	95 (43.2%)	125 (56.8%)	0.614	8
**Negative perceptions**					
I do not believe that vaccines are effective	254 (32.2%)	114 (44.9%)	140 (55.1%)	0.655	6
I believe that vaccines may have dangerous side effects	400 (50.6%)	161 (40.3%)	239 (59.8%)	0.755	4
I believe that flu vaccine causes flu and fever	407 (51.5%)	146 (35.9%)	261 (64.1%)	0.760	3
I believe I will not get flu	270 (34.2%)	131 (48.5%)	139 (51.5%)	0.668	5
Flu is seasonal, it will recover on its own	390 (49.4%)	143 (36.7%)	247 (63.3%)	0.762	2
**Lack of recommendation**					
I was not recommended to get a flu vaccination by my doctor	407 (51.5%)	170 (41.8%)	237 (58.2%)	0.775	1
**Religious and cultural restrictions**					
I don’t want to get a flu vaccination because of religious reasons	230 (29.1%)	145 (63%)	85 (37%)	0.626	9
I don’t want to get a flu vaccination because of cultural reasons	214 (27.1%)	139 (65%)	75 (35%)	0.619	10

**Notes.**

Only agreed responses were included.

a % Agreement: Percentage of participants who agreed to the statement (score of 4–5).

b Relative index.

c Barriers and perceptions to influenza vaccine uptake were ranked as per RII.

### Willingness to receive influenza vaccine

The mean willingness score among study participants was 3.54 ±  1.57 (Table 4). About one-third (*n* = 289, 36.6%) of study participants were willing to get vaccinated every year. However, the majority of the respondents (*n* = 545, 69%) were willing to receive the flu shot on their doctor’s recommendations, and 46.1% agreed to get the vaccine if provided free of cost at the university. The vaccine is available in intramuscular injection and intra-nasal spray dosage forms in various parts of the world. However, only the intramuscular route of the flu vaccine is available in Saudi Arabia. Surprisingly, most students (*n* = 496, 62.8%) were inclined to get a flu vaccination via intramuscular route compared to intra-nasal spray (*n* = 201, 25.4%). Willingness status was further stratified as vaccine acceptor (*n* = 289, 36.6%), vaccine rejecter (*n* = 131, 16.6%), and vaccine ambivalent (*n* = 370, 46.8%). Moreover, the majority (*n* = 277, 77.6%) of the non-vaccinated participants reported a poor level of willingness. However, half of the study population (*n* = 390, 49.4%) had a moderate level of willingness to receive the flu vaccine. There was a positive correlation (0.001*) between the knowledge and willingness score for the flu vaccine.

**Table 4:** Willingness to receive influenza vaccine (*N* = 790).

**Item**	Total respondents^*^n (%)	**Health sciences (*n* = 241)**	**Non-health sciences (*n* = 549)**	*p*-value	**Vaccinated participants (*n* = 240)**	**Non-vaccinated participants (*n* = 549)**	*p*-value
**Willingness to get vaccine and justification**							
I will regularly get a flu vaccine every year (vaccine acceptor)	289 (36.6%)	97 (33.6%)	192 (66.4%)	0.264	131 (45.3%)	158 (54.7%)	**<0.001**
I will get a flu vaccine if myself or any of my family member got flu	199 (25.2%)	38 (19.1%)	161 (80.9%)	**<0.001**	72 (36.2%)	127 (63.8%)	**0.044**
I will get a flu vaccine only if my doctor recommends me	545 (69%)	152 (27.9%)	393 (72.1%)	**0.003**	161 (29.5%)	384 (70.5%)	0.380
I will get a flu vaccine if yearly flu vaccination is made compulsory in National Immunization Program	362 (45.8%)	100 (27.6%)	262 (72.4%)	**<0.001**	110 (30.4%)	252 (69.6%)	**0.001**
I will get a flu vaccine if it is provided in university campus free of cost	364 (46.1%)	123 (33.8%)	241 (66.2%)	0.137	131 (36%)	233 (64%)	**0.002**
**Vaccine type preference**							
I will get a flu vaccination via intramuscular (IM) shot (injection)	496 (62.8%)	179 (36.1%)	317 (63.9%)	**<0.001**	149 (30%)	347 (70%)	0.712
I will get a flu vaccination via intranasal mist/spray	201 (25.4%)	80 (39.8%)	121 (60.2%)	**<0.001**	81 (40.3%)	120 (59.7%)	**<0.001**
**Mean willingness score**							
Mean ± SD	2.94 ± 1.45	3.14 ± 1.31	2.85 ± 1.50	**<0.001**	3.37 ± 1.61	2.74 ± 1.32	**<0.001**
Willingness categories^**^				**<0.001**			**<0.001**
Good willingness	43 (5.4%)	7 (16.3%)	36 (83.7%)		23 (53.5%)	20 (46.5%)	
Moderate willingness	390 (49.4%)	151 (38.7%)	239 (26.3%)		138 (35.4%)	252 (64.6%)	
Poor willingness	357 (45.2%)	88 (24.6%)	269 (75.4%)		80 (22.4%)	277 (77.6%)	

**Notes.**

* Participants agreeing to the statement.

** Categories according to the willingness score.

*p*-values were estimated using *χ*^2^ and *t*-tests with a significance level of 0.05; these statistically significant values and recorded in bold fonts in this table.

### Determinants of influenza vaccine uptake

In Table 5, the majority of the population (*n* = 549, 69.5%) did not take the vaccine shot before winter. In binary analysis, age (22–25 years: OR: 0.730, *p* = 0.044), study field (non-health sciences: OR: 1.715, *p* = 0.001), poor knowledge score (OR: 2.021, *p* = 0.010), and poor willingness level (OR: 2.049, *p* <0.001) were found to be associated with reduced vaccine uptake. Adjusted regression analysis also identified similar factors associated with the reduced vaccine uptake.

**Table 5:** Predictors of reduced vaccine uptake.

**Variable**	**Bivariate analysis**			**Multivariate analysis**		
	**OR**	*p*-value	**95% CI**	**OR**	**p-value**	**95% CI**
**Age categories**	
18–21 years	ref	–	–	Ref	–	–
22–25 years	0.730	**0.044**	0.537–0.992	0.208	**0.038**	0.047–0.918
≥ 25 years	0.259	0.072	0.060–1.127	0.157	**0.015**	0.036–0.693
**Gender**	
Male	ref	–	–	–	–	–
Female	0.860	0.328	0.635–1.164	–	–	–
**Marital status**	
Single	ref	–	–	–	–	–
Married	0.868	0.493	0.579–1.302	–	–	–
**Study field**	
Health sciences	ref	**–**	–	–	–	–
Non-health sciences	1.715	**0.001**	1.246–2.360	0.661	**0.015**	0.475–0.922
Poor knowledge score	2.021	**0.010**	1.181–3.459	1.786	**0.039**	1.030–3.094
Poor willingness level	2.049	**<0.001**	1.494–2.811	2.014	**<0.001**	1.458–2.781

**Notes.**

*p*-values >0.05 were excluded from the multivariate analysis.

Odds Ratio (OR) and Confidence Interval (CI) have been rounded off staistically signficant values were recorded in bold in this table.

## Discussion

The educational institutes are required to provide the primary platform to their students to avail all necessary health awareness in addition to academics. Since the students contribute a large proportion of the country’s population, their participation in vaccination campaigns is an impetus to achieve herd immunity against vaccine-preventable diseases. To the best of our knowledge, this is the first cross-sectional analysis evaluating the flu vaccination rate, knowledge, and barriers among health-sciences and non-health-sciences students from a Saudi University.

Despite the recommendation of the annual vaccine against influenza, the vaccine uptake rate remains considerably low, especially among students. Generally, students consider themselves healthy individuals who are less likely to contract the infection and have a low vaccination rate. This survey reported that only one-third of the students were vaccinated before the arrival of the winter season. These results corroborate the findings of other studies (Choucair et al., 2021; Benjamin & Bahr, 2016; Endrich, Blank & Szucs, 2009; Blank, Schwenkglenks & Szucs, 2009). However, these findings are evidently lower than the previously conducted studies in Saudi Arabia (Rogers, Bahr & Benjamin, 2018; Alkathlan et al., 2020; Alshammari et al., 2014), which might be possible due to a wide disparity in study population and methodology.

Only 20.4% of students demonstrated good knowledge regarding flu vaccination and knowledge score was comparatively better among HS students (88, 54.7%). However, it is worth mentioning that a large proportion of HS students recorded incorrect responses for some items. Since influenza vaccines are quadrivalent and provide protection against four viral strains, 30.1% of HS students responded that the influenza vaccine provides coverage against all flu-causing viral strains. Though vaccinated participants and those from HS disciplines demonstrated better knowledge, educational interventions must be initiated regardless of the study disciplines and vaccination status. Unsatisfactory vaccination rates and poor knowledge have also been reported in another study from Saudi Arabia (Alhawsawi et al., 2020).

This study identified 11 barriers to vaccine uptake among students, including five negative perceptions of the flu vaccine. These barriers might be addressed through awareness and educational campaigns at the university level. The proportion of these barriers was more profound among non-vaccinated students. They have concerns about vaccine efficacy, safety, and side effects, and these findings are consistent with the previous studies (Woolf, Chapman & Lee, 2021; Taylor et al., 2020; Fisher et al., 2020; Ali et al., 2018; Ru-Chien & Neuzil, 2004). We have classified the barriers into five broad categories including affordability, accessibility, negative perceptions, lack of recommendation, and cultural or religious restrictions. The lack of recommendation from the healthcare professionals was found as a top-ranked barrier to flu vaccination. These results necessitate the need for target interventions on health professionals so they could guide their patients on the importance of flu vaccines. Moreover, arranging the health screening camps at the university level will aid to overcome these barriers and improve the vaccination rates among students. The lack of recommendations for flu vaccines from healthcare professionals has also been reported in other studies (Bovier et al., 2001). It is important to note that 69% of participants agreed to get the flu shots if their physicians would make such recommendations. This could be turned into an effective facilitator, improving the overall vaccination status. The importance of this facilitator has also been discussed in another study (Ru-Chien & Neuzil, 2004). Educating the population about the significance of the influenza vaccine directly influences the willingness of the individuals to get vaccinated (Bednarczyk et al., 2015). Awareness campaigns can be arranged at the institutional level, via media, and through other online resources. Educational activities also need to address the negative perceptions, misleading narratives, and myths prevailing among students. Awareness of the students will positively influence the decision-making skills of the emerging adult population.

Approximately, one-third (36.6%) of the study population were willing to get the flu vaccine every year. However, this proportion increased to 46.1% if the flu vaccines would be provided free of cost on-campus. Two-thirds of the participants were willing to get the flu shot if it is recommended by their doctors. The low vaccination rate in this study is also associated with a lack of awareness about the whereabouts of the vaccination centers, as one-fourth of the study population reported that they do not know where to get the flu shots. An explanation for this association could be that those who knew where to get their flu shot were most likely to get vaccinated. In addition, non-vaccinated participants were more willing to receive the flu vaccine in the future. On the other hand, vaccinated participants demonstrated a lesser intention to take the vaccine again. The accessibility of the vaccine is a pivotal facilitator in achieving a high vaccination rate. Considering the poor health-seeking behaviour of the students, accessibility of the vaccines in the universities at the micro-level will aid to improve the vaccine uptake in this population. Lower vaccine hesitancy was reported (16.6%) as compared to a study conducted by Alzeer et al. (2021), which reported 42% vaccine hesitancy among the participants.

The increased age, study discipline, poor knowledge, and willingness for vaccines were found to be associated with reduced vaccine uptake. This study did not demonstrate any association between knowledge and willingness to receive the vaccine (*p* = 0.349). The results of previous investigations showed that only good knowledge is not enough to encourage vaccine uptake and attitudes about the value and risk, as well as negative perceptions also negate the decision of getting influenza vaccine (Black et al., 2015; O’Reilly, Cran & Stevens, 2005; Romine, Barrow & Folk, 2013; Martinello, Jones & Topal, 2003).

Students who were vaccinated and from healthcare disciplines had higher knowledge of vaccine uptake which might be attributed to their attitude towards the importance of vaccination. In addition, females demonstrated higher knowledge as compared to males. Since students having high knowledge scores are more likely to receive the flu vaccination, this hypothesis was further tested. Our analysis showed that poor knowledge and willingness were associated with reduced vaccine uptake. In addition to the demographics, there is a dire need to evaluate some other factors influencing the decision-making behaviour of students for vaccination such as myths and anti-vaxxers campaigns on social media. The improvement of the knowledge is proportionate to the willingness to take vaccines, thereby increasing the vaccination rate. In this context, the first and foremost priority of the educational institutes should be the evaluation of the knowledge and attitude of their students towards the flu vaccine.

Encouraging the university-wide uptake of flu vaccine, various public-level interventions are also required. Incentives (coupons, give aways, etc.) might play an additional motivating role and encourage the students to get the flu jab. As discussed earlier, the provision of free vaccines at the institutional or national level will significantly contribute to achieving the targeted vaccine coverage. It is pertinent to mention that the motivation of the students to take the vaccine is potentially transmittable to their family, friends, and relatives. Moreover, the students from healthcare disciplines could play an additional role in the vaccination campaign by sensing it as their professional and ethical duty by considering the fact that they will be healthcare professionals in the future. An efficient way of increasing the vaccine coverage is to introduce interventions that would specifically target the vaccine-hesitant and ambivalent population. Understanding the perception of students and addressing the knowledge gaps by introducing educational programs will boost the awareness of the students, especially those from non-health science disciplines.

The present study is limited by self-reporting of vaccine uptake status which could be a potential bias. Additionally, convenient sampling was used which limits the generalizability of the outcomes. There is a probability that the students who participated in the survey could be different from those who did not participate. This could lead to over-estimate and under-estimate of the vaccination rate, barriers, and willingness score. Furthermore, due to the cross-sectional study design, we were unable to establish causal inferences even though the association between the variables might exist. This study was single-centered and might not be extrapolated to the students of other universities. Moreover, the sampled population may have the bias of having the population who was less inclined to get vaccinated. Nevertheless, this study is strengthened by the large sample size and the first representation of the flu vaccine status among students from health and non-health science disciplines in a public university in Saudi Arabia. The implications of this study can be considered for all universities, as public and private educational institutes follow similar policies and procedures to the Ministry of Education. Moreover, a heterogeneous study population is another strength of this survey.

## Conclusions

This study underscored the unsatisfactory flu vaccination rate among university students. The study participants demonstrated poor knowledge and were less inclined to receive the flu vaccine in the future. Lack of recommendation from the physicians, negative perceptions towards the flu vaccine, and difficult accessibility were found as significant barriers to the vaccine uptake. The knowledge, barriers, and willingness widely varied across students from various disciplines. Lower than recommended vaccination coverage is a global concern for vaccine-preventable influenza. The vaccination rate in this study is expected to be improved as per the willingness of students. A multidimensional approach needs to be adopted to enhance the vaccination coverage such as educational campaigns, awareness seminars, and safe and free provision of vaccines at the university level. Continued education and communication play a vital role in changing the perception over time, hence, encouraging students to achieve high vaccine coverage. Moreover, future research could focus on multi-institutional qualitative studies including on the safety and efficacy of the influenza vaccine.

##  Supplemental Information

10.7717/peerj.13959/supp-1 Supplemental Information 1 Raw data

10.7717/peerj.13959/supp-2 Supplemental Information 2 Arabic Questionnaire

10.7717/peerj.13959/supp-3 Supplemental Information 3 Questionnaire

## Additional Information and Declarations

### Data Availability

The following information was supplied regarding data availability: The raw measurements are available as a Supplementary File.

## References

[ref-1] Abu-Gharbieh E, Fahmy S, Rasool BA, Khan S (2010). Influenza vaccination: healthcare workers attitude in three Middle East countries. International Journal of Medical Sciences.

[ref-2] Al-Tawfiq JA (2012). Willingness of health care workers of various nationalities to accept H1N1 (2009) pandemic influenza A vaccination. Annals of Saudi Medicine.

[ref-3] Alhawsawi MM, Alghamdi AA, Alzayed BM, Binmugren HM, Alshehri RA, Abusalih HH (2020). Knowledge barriers and uptake of influenza vaccine among non-health college students at Princess Nourah Bint Abdulrahman University, Riyadh, Saudi Arabia. Journal of Public Health Research.

[ref-4] Ali I, Ijaz M, Rehman IU, Rahim A, Ata H (2018). Knowledge, and attitude, and awareness, and barriers toward influenza vaccination among medical doctors at tertiary care health settings in Peshawar, Pakistan–a cross-sectional study. Frontiers in Public Health.

[ref-5] Ali S, Khakoo R, Fisher M, Hobbs GR (2007). An assessment of influenza vaccination among health profession students. Scandinavian Journal of Infectious Diseases.

[ref-6] Alkathlan M, Khalil R, Alhemaidani MF, Alaed GH, Almutairi SM, Almalki HA, Alghofaili RH, Al-Wutayd O (2020). Trends, uptake, and predictors of influenza vaccination among healthcare practitioners during the COVID-19 pandemic flu season and the following season (2021) in Saudi Arabia. Journal of Multidisciplinary Healthcare.

[ref-7] Alshammari TM, AlFehaid LS, AlFraih JK, Aljadhey HS (2014). Health care professionals’ awareness of knowledge about and attitude to influenza vaccination. Vaccine.

[ref-8] Alzeer AA, Alfantoukh LA, Theneyan A, Eid FB, Almangour TA, Alshememry AK, Alhossan AM (2021). The influence of demographics on influenza vaccine awareness and hesitancy among adults visiting educational hospital in Saudi Arabia. Saudi Pharmaceutical Journal.

[ref-9] Bali NK, Ashraf M, Ahmad F, Khan UH, Widdowson M-A, Lal RB, Koul PA (2013). Knowledge, attitude, and practices about the seasonal influenza vaccination among healthcare workers in Srinagar, India. Influenza and Other Respiratory Viruses.

[ref-10] Balkhy HH, Memish ZA, Bafaqeer S, Almuneef MA (2004). Influenza a common viral infection among Hajj pilgrims: time for routine surveillance and vaccination. Journal of Travel Medicine.

[ref-11] Bednarczyk RA, Chu SL, Sickler H, Shaw J, Nadeau JA, McNutt L-A (2015). Low uptake of influenza vaccine among university students: evaluating predictors beyond cost and safety concerns. Vaccine.

[ref-12] Benjamin SM, Bahr KO (2016). Barriers associated with seasonal influenza vaccination among college students. Influenza Research and Treatment.

[ref-13] Black CL, Yue X, Ball SW, Donahue SMA., Izrael D, de Perio MA, Laney AS, Williams WW, Lindley MC, Graitcer SB, Lu P-J, Bridges CB, DiSogra C, Sokolowski J, Walker DK, Greby SM (2015). Influenza vaccination coverage among health care personnel—United States, 2014–15 influenza season. Morbidity and Mortality Weekly Report.

[ref-14] Blank PR, Schwenkglenks M, Szucs TD (2009). Vaccination coverage rates in eleven European countries during two consecutive influenza seasons. Journal of Infection.

[ref-15] Bovier PA, Chamot E, Bouvier Gallacchi M, Loutan L (2001). Importance of patients’ perceptions and general practitioners’ recommendations in understanding missed opportunities for immunisations in Swiss adults. Vaccine.

[ref-16] Buchman TG, Simpson SQ, Sciarretta KL, Finne KP, Sowers N, Collier M, Chavan S, Do R, Lin C, Oke I, Rhodes KE, Santhosh A, Sandhu AT, Chu S, Patel SA, Disbrow GL, Bright RA, MaCurdy TE, Kelman JA (2021). Seasonal influenza vaccination is associated with reduced risk of death among Medicare beneficiaries*. Vaccine.

[ref-17] Choucair K, El Sawda J, Assaad S, El Chakhtoura NG, Hassouna H, Sidani N, Yasmin M, Rteil A, Kanj SS, Kanafani ZA (2021). Knowledge, perception, attitudes and behavior on influenza immunization and the determinants of vaccination. Journal of Epidemiology and Global Health.

[ref-18] Endrich MM, Blank PR, Szucs TD (2009). Influenza vaccination uptake and socioeconomic determinants in 11 European countries. Vaccine.

[ref-19] Eppes C, Wu A, You W, Cameron KA, Garcia P, Grobman W (2013). Barriers to influenza vaccination among pregnant women. Vaccine.

[ref-20] Fisher KA, Bloomstone SJ, Walder J, Crawford S, Fouayzi H, Mazor KM (2020). Attitudes toward a potential SARS-CoV-2 vaccine: a survey of US adults. Annals of Internal Medicine.

[ref-21] GBD 2017 Influenza Collaborators (2019). Mortality, morbidity, and hospitalisations due to influenza lower respiratory tract infections, 2017: an analysis for the Global Burden of Disease Study 2017. The Lancet Respiratory Medicine.

[ref-22] Hamdan MB, Singh S, Polavarapu M, Jordan TR, Melhem NM (2021). COVID-19 vaccine hesitancy among university students in Lebanon. Epidemiology & Infection.

[ref-23] Hayward AC, Fragaszy EB, Bermingham A, Wang L, Copas A, Edmunds WJ, Ferguson N, Goonetilleke N, Harvey G, Kovar J, Lim MSC, McMichael A, Millett ERC, Nguyen-Van-Tam JS, Nazareth I, Pebody R, Tabassum F, Watson JM, Wurie FB, Johnson AM, Zambon M (2014). Comparative community burden and severity of seasonal and pandemic influenza: results of the Flu Watch cohort study. The Lancet Respiratory Medicine.

[ref-24] Kim H, Webster RG, Webby RJ (2018). Influenza virus: dealing with a drifting and shifting pathogen. Viral Immunology.

[ref-25] Lee SI, Aung EM, Chin IS, Hing JW, Mummadi S, Palaniandy GD, Jordan R (2012). Factors affecting medical students’ uptake of the 2009 pandemic influenza A (H1N1) vaccine. Influenza Research and Treatment.

[ref-26] MacDonald NE (2015). Vaccine hesitancy: definition, scope and determinants. Vaccine.

[ref-27] Martinello RA, Jones L, Topal JE (2003). Correlation between healthcare workers’ knowledge of influenza vaccine and vaccine receipt. Infection Control & Hospital Epidemiology.

[ref-28] Merrill RM, Kelley TA, Cox E, Layman AB, Layton BJ, Lindsay R (2010). Factors and barriers influencing influenza vaccination among students at Brigham Young University. Medical Science Monitor.

[ref-29] Milunic SL, Quilty JF, Super DM, Noritz GH (2010). Patterns of influenza vaccination among medical students. Infection Control & Hospital Epidemiology.

[ref-30] Nichol KL, D’Heilly S, Ehlinger EP (2008). Influenza vaccination among college and university students: impact on influenzalike illness, health care use, and impaired school performance. Archives of Pediatrics & Adolescent Medicine.

[ref-31] Oakley S, Bouchet J, Costello P, Parker J (2021). Influenza vaccine uptake among at-risk adults (aged 16–64 years) in the UK: a retrospective database analysis. BMC Public Health.

[ref-32] O’Reilly FW, Cran GW, Stevens AB (2005). Factors affecting influenza vaccine uptake among health care workers. Occupational Medicine.

[ref-33] Paget J, Spreeuwenberg P, Charu V (2019). Global mortality associated with seasonal influenza epidemics: New burden estimates and predictors from the GLaMOR Project. Journal of Global Health.

[ref-34] Rodríguez-Blanco N, Tuells J (2019). Knowledge and attitudes about the flu vaccine among pregnant women in the valencian community (Spain). Medicina.

[ref-35] Rogers CJ, Bahr KO, Benjamin SM (2018). Attitudes and barriers associated with seasonal influenza vaccination uptake among public health students; a cross-sectional study. BMC Public Health.

[ref-36] Romine WL, Barrow LH, Folk WR (2013). Exploring secondary students’ knowledge and misconceptions about influenza: development, validation, and implementation of a multiple-choice influenza knowledge scale. International Journal of Science Education.

[ref-37] Ru-Chien CHI, Neuzil KM (2004). The association of sociodemographic factors and patient attitudes on influenza vaccination rates in older persons. The American Journal of the Medical Sciences.

[ref-38] Ryan KA, Filipp SL, Gurka MJ, Zirulnik A, Thompson LA (2019). Understanding influenza vaccine perspectives and hesitancy in university students to promote increased vaccine uptake. Heliyon.

[ref-39] Salem S, Miligi E, Alanazi HH, Alanazi NA, Alanazi AA (2019). Knowledge and limitations associated with the uptake of seasonal influenza vaccine among nursing students. International Journal of Novel Research in Healthcare and Nursing.

[ref-40] Salman M, Mustafa ZU, Asif N, Zaidi HA, Hussain K, Shehzadi N, Khan TM, Saleem Z (2020). Knowledge, attitude and preventive practices related to COVID-19: a cross-sectional study in two Pakistani university populations. Drugs & Therapy Perspectives.

[ref-41] Silva J, Bratberg J, Lemay V (2021). COVID-19 and influenza vaccine hesitancy among college students. Journal of the American Pharmacists Association.

[ref-42] Srichan P, Apidechkul T, Tamornpark R, Yeemard F, Khunthason S, Kitchanapaiboon S, Wongnuch P, Wongphaet A, Upala P (2020). Knowledge, attitudes and preparedness to respond to COVID-19 among the border population of northern Thailand in the early period of the pandemic: a crosssectional study. WHO South-East Asia Journal of Public Health.

[ref-43] Taylor S, Landry CA, Paluszek MM, Groenewoud R, Rachor GS, Asmundson GJG (2020). A proactive approach for managing COVID-19: the importance of understanding the motivational roots of vaccination hesitancy for SARS-CoV2. Frontiers in Psychology.

[ref-44] WHO (2019a). Influenza (seasonal). World Health Organization. https://www.who.int/news-room/fact-sheets/detail/influenza-(seasonal).

[ref-45] WHO (2019). Ten threats to global health in 2019.

[ref-46] Woolf SH, Chapman DA, Lee JH (2021). COVID-19 as the leading cause of death in the United States. JAMA.

