# Supplementary material for: Evaluation of knowledge and barriers of influenza vaccine uptake among university students in Saudi Arabia; a cross-sectional analysis
Source: PeerJ. 2022 Sep 28;10:e13959. doi: 10.7717/peerj.13959 (PMC9526417; doi:10.7717/peerj.13959)
Supplement: Supplemental Information 2 [file peerj-10-13959-s002.pdf]

Data Collection form

Knowledge, Perceptions and Barriers of influenza vaccine uptake among university students in Jouf, Saudia Arabia.

المعرفة والتصورات وحواجز تناول لقاح الأنفلونزا بين طلاب الجامعه في منطقة الجوف بالمملكة العربية السعودية.

Part 1: Demographics

**Student ID:** الرقم الجامعي	**Gender:** الجنس<br>o Female
**Age:** (العمر)	**Marital status:** (الحالة الزوجية)<br>o Single (أعزب)<br>o Married (متزوج)<br>**If married, please mention how many children** (إذا كنت متزوج ، آمل ذكر عدد الأطفال):
**No. of years in university**<br>(عدد السنوات في الجامعة)	**Field of education**(مجال الدراسة في الجامعة):<br>o Pharmacy college (كلية الصيدلة)<br>o Preparatory (تحضيري)<br>o Physical therapy (العلاج الطبيعي)<br>o Business administration (إدارة أعمال)<br>o Education college (كلية التربية)
**Routinely make own hospital appointment**<br>(عادة، تقوم بحجز موعدك بنفسك في المستشفى):<br>o Yes (نعم)<br>o No (لا)	**Received flu vaccination anytime in childhood:**<br>(في طفولتك، هل تم تطعيمك ضد الأنفونزا)<br>o Yes (نعم)<br>o No (لا)
**Received flu vaccination before joining university:**<br>هل تم تطعيمك ضد الأنفونزا قبل إنضمامك للجامعة؟)<br>o Yes (نعم)<br>o No (لا)	**Received flu vaccination in the past 3 months (before arrival of winter)**<br>هل تطعمت ضد الإنفونزا في الأشهر الثلاث السابقة (قبل دخول فصل الشتاء)؟<br>o Yes (نعم)<br>o No (لا)

Scanned with CamScanner

Part 2: Knowledge regarding Flu vaccination

(المعرفة بشأن التطعيم ضد الانفلونزا)

Statement(العبارات)	True (صحيح)	False (خطأ)
All persons aged 6 months and above should get influenza vaccination annually<br>الأشخاص الذين يبلغون من العمر ٦ أشهر و أعلى ، يجب عليهم الحصول على لقاح الأنفلونزا سنوياً		
Influenza vaccination causes mild flu like symptoms<br>يسبب التطعيم ضد الانفلونزا أعراض خفيفة شبيهة بأعراض الانفلونزا		
Being vaccinated reduces the severity and duration of flu<br>التطعيم يقلل من شدة ومدة الانفلونزا		
Being vaccinated, improves immunity<br>التطعيم ، يحسن المناعة		
Infants and immuno-compromised population cannot get influenza vaccination<br>لا يمكن للأطفال الرضع والأشخاص ضعيفي المناعة الحصول على لقاح الأنفلونزا		
The complications of influenza can be severe leading to absence from schools and workplace, effecting quality of work<br>يمكن أن تكون مضاعفات الأنفلونزا شديدة تؤدي إلى الغياب عن المدارس ومكان العمل ، مما يؤثر على جودة العمل		
Severe influenza can lead to hospitalization and even death<br>يمكن أن تؤدي الأنفلونزا الحادة إلى دخول المستشفى وحتى الموت		
Influenza vaccine provides coverage for all types of strains that cause flu<br>يوفر لقاح الأنفلونزا تغطية لجميع أنواع السلالات التي تسبب الإنفلونزا		
Influenza vaccine reduces the severity and duration of flu for all types of strains<br>لقاح الأنفلونزا يقلل من شدة ومدة الإنفلونزا لجميع أنواع السلالات المسببة لها		
Influenza vaccine is not effective if I already got flu<br>لقاح الأنفلونزا غير فعال إذا أصبت بالفعل بالإنفلونزا		
There are two types of influenza vaccine; intramuscular shot, intra nasal spray<br>هناك نوعان من طرق إعطاء لقاح الأنفلونزا ؛ حقنة بالعضل و رذاذ داخل الأنف		
The intramuscular influenza "shot" vaccine contains inactivated (killed) virus<br>لقاح الأنفلونزا العضلي يحتوي على فيروس غير نشط (خامل)		
The intranasal influenza "spray" vaccine (FluMist) contains live attenuated virus<br>يحتوي لقاح "الرذاذ" للأنفلونزا داخل الأنف (FluMist) على فيروس موهّن حي		

Scanned with CamScanner

Part 3: Perceptions and Barriers to receive flu vaccination

تصورات وحواجز لتلقي التطعيم ضد الانفلونزا

**Statements** العبارات	**Strongly Agree** أوافق بشدة	**Agree** أوافق	**Neutral** محايد	**Disagree** لا أوافق	**Strongly disagree** لا أوافق بشدة
Vaccines are expensive<br>اللقاحات غالية الثمن					
I do not have time to get a flu vaccination<br>ليس لدي الوقت للحصول على تطعيم ضد الانفلونزا					
I do not know where to receive a flu vaccination<br>أنا لا أعرف من أين احصل على التطعيم ضد الانفلونزا					
I do not believe that vaccines are effective<br>لا أعتقد أن اللقاحات (التطعيمات) فعالة					
I believe that vaccines may have dangerous side effects<br>أعتقد أن اللقاحات قد يكون لها آثار جانبية خطيرة					
I believe that flu vaccine causes flu and fever<br>أعتقد أن لقاح الأنفلونزا يسبب الأنفلونزا والحمى (ارتفاع درجة حرارة الجسم)					
I believe I will not get flu<br>أعتقد أنني لن أصاب بالأنفلونزا					
I was not asked to get a flu vaccination by my doctor<br>لم يطلب مني طبيبي أن احصل على تطعيمة الإنفلونزا					
Flu is seasonal, It will recover on its own<br>عدوى الأنفلونزا موسمية ، وسوف اتعافى منها تلقائياً					
I don't want to get a flu vaccination because of religious reasons<br>لا أريد الحصول على لقاح ضد الأنفلونزا لأسباب تتعلق بديني					
I don't want to get a flu vaccination because of cultural reasons<br>لا أريد الحصول على اللقاح ضد الأنفلونزا لأسباب ثقافية					

Part 4: Preparedness/ willingness to receive influenza vaccination

الاستعداد / الرغبة لتلقي لقاح الأنفلونزا

Statement العبارات	Yes نعم	No لا	Don't know لا أعرف
I will regularly get a flu vaccine every year<br>سأحصل بانتظام على لقاح الأنفلونزا كل عام			
I will get a flu vaccine only if myself or any of my family member got flu<br>سوف أحصل على لقاح الأنفلونزا فقط إذا أصبت أنا أو أحد أفراد أسرتي بالأنفلونزا			
I will get a flu vaccine only if my doctor recommends me<br>سأحصل على لقاح الأنفلونزا فقط إذا أوصى طبيبي بذلك			

Scanned with CamScanner

I will get a flu vaccine only if yearly flu vaccination is made compulsory in National Immunization Program<br>سأحصل على لقاح الأنفلونزا فقط إذا أصبح التطعيم السنوي ضد الإنفلونزا إلزاميًا في البرنامج الوطني للتحصين ضد الأمراض المعدية			
I will get a flu vaccine if it is provided in university campus free of cost<br>سأحصل على لقاح الأنفلونزا إذا تم توفيره في الحرم الجامعي مجانًا			
I will get a flu vaccination via intramuscular shot (injection)<br>سأحصل على لقاح الأنفلونزا عن طريق الحقن بالعضل			
I will get a flu vaccination via intranasal mist/ spray<br>سأحصل على التطعيم ضد الأنفلونزا عن طريق رذاذ / رش داخل الأنف			
I will not get a flu vaccine in any case<br>لن أحصل على لقاح الأنفلونزا على أي حال			

Scanned with CamScanner
