# Supplementary material for: Evaluation of knowledge and barriers of influenza vaccine uptake among university students in Saudi Arabia; a cross-sectional analysis
Source: PeerJ. 2022 Sep 28;10:e13959. doi: 10.7717/peerj.13959 (PMC9526417; doi:10.7717/peerj.13959)
Supplement: Supplemental Information 3 [file peerj-10-13959-s003.docx]

**Knowledge, Perceptions and Barriers of influenza vaccine uptake among university students in Jouf, Saudi Arabia**

**Section I: Demographics**

1. **Gender**
   1. Male
   2. Female
2. **Age. ___________**
3. **Age Category**
   1. 18-21 years
   2. 22-25 years
   3. ≥ 25 years
4. **Marital status**
   1. Single
   2. Married
5. **Number of Years in University ___________**
6. **Field of Education**
   1. Health Sciences
   2. Non-Health Sciences
7. **Routinely make own hospital appointments**
   1. Yes
   2. No
8. **Received flu vaccination anytime in childhood**
   1. Yes
   2. No
   3. Maybe
9. **Received flu vaccination before joining university**
   1. Yes
   2. No
10. **Received flu vaccination in the past 3 months (before arrival of winter)**
    1. Yes
    2. No

**Section II: Knowledge regarding Flu vaccination**

1. All persons aged 6 months and above should get influenza vaccination annually
   1. True
   2. False
2. Influenza vaccination causes mild flu like symptoms
   1. True
   2. False
3. Being vaccinated reduces the severity and duration of flu
   1. True
   2. False
4. Being vaccinated, improves immunity
   1. True
   2. False
5. Infants and immuno-compromised population cannot get influenza vaccination
   1. True
   2. False
6. The complications of influenza can be severe leading to absence from schools and work place, effecting quality of work
   1. True
   2. False
7. Severe influenza can lead to hospitalization an even death
   1. True
   2. False
8. Influenza vaccine provides coverage for all types of strains that cause flu
   1. True
   2. False
9. Influenza vaccine reduces the severity and duration of flu for all types of strains
   1. True
   2. False
10. Influenza vaccine is not effective if I already got flu
    1. True
    2. False
11. There are two types of influenza vaccine; intramuscular shot, intra nasal spray
    1. True
    2. False
12. The intramuscular influenza "shot" vaccine contains inactivated (killed) virus
    1. True
    2. False
13. The intranasal influenza "spray" vaccine (FluMist) contains live attenuated virus
    1. True
    2. False

**Section III: Perceptions and Barriers to receive flu vaccination**

1. **Vaccines are expensive**
   1. Strongly Agree
   2. Agree
   3. Neutral
   4. Disagree
   5. Strongly Disagree
2. **I do not have time to get a flu vaccination**
   1. Strongly Agree
   2. Agree
   3. Neutral
   4. Disagree
   5. Strongly Disagree
3. **I do not know where to receive a flu vaccination**
   1. Strongly Agree
   2. Agree
   3. Neutral
   4. Disagree
   5. Strongly Disagree
4. **I do not believe that vaccines are effective**
   1. Strongly Agree
   2. Agree
   3. Neutral
   4. Disagree
   5. Strongly Disagree
5. **I believe that vaccines may have dangerous side effects**
   1. Strongly Agree
   2. Agree
   3. Neutral
   4. Disagree
   5. Strongly Disagree
6. **I believe that flu vaccine causes flu and fever**
   1. Strongly Agree
   2. Agree
   3. Neutral
   4. Disagree
   5. Strongly Disagree
7. **I believe I will not get flu**
   1. Strongly Agree
   2. Agree
   3. Neutral
   4. Disagree
   5. Strongly Disagree
8. **I was not asked to get a flu vaccination by my doctor**
   1. Strongly Agree
   2. Agree
   3. Neutral
   4. Disagree
   5. Strongly Disagree
9. **Flu is seasonal, it will recover on its own**
   1. Strongly Agree
   2. Agree
   3. Neutral
   4. Disagree
   5. Strongly Disagree
10. **I don’t want to get a flu vaccination because of religious reasons**
    1. Strongly Agree
    2. Agree
    3. Neutral
    4. Disagree
    5. Strongly Disagree
11. **I don’t want to get a flu vaccination because of cultural reasons**
    1. Strongly Agree
    2. Agree
    3. Neutral
    4. Disagree
    5. Strongly Disagree

**Section IV: Preparedness/ willingness to receive influenza vaccination**

1. **I will regularly get a flu vaccine every year**
   1. Yes
   2. No
   3. Do not know
2. **I will get a flu vaccine only if myself or any of my family member got flu**
   1. Yes
   2. No
   3. Do not know
3. **I will get a flu vaccine only if my doctor recommends me**
   1. Yes
   2. No
   3. Do not know
4. **I will get a flu vaccine only if yearly flu vaccination is made compulsory in National Immunization Program**
   1. Yes
   2. No
   3. Do not know
5. **I will get a flu vaccine if it is provided in university campus free of cost**
   1. Yes
   2. No
   3. Do not know
6. **I will get a flu vaccination via intramuscular shot (injection)**
   1. Yes
   2. No
   3. Do not know
7. **I will get a flu vaccination via intranasal mist/ spray**
   1. Yes
   2. No
   3. Do not know
8. **I will not get a flu vaccine in any case**
   1. Yes
   2. No
   3. Do not know
